# Discerning undifferentiated anxiety from syndromal anxiety in acute-phase schizophrenia

**DOI:** 10.1186/s12991-020-00277-4

**Published:** 2020-04-15

**Authors:** Kalai Naidu, Werdie van Staden, Lizelle Fletcher

**Affiliations:** 1 Department of Psychiatry, University of Pretoria, Pretoria, South Africa; 2 Centre for Ethics and Philosophy of Health Sciences, Faculty of Health Sciences, University of Pretoria, Arcadia, Private Bag X323, Pretoria, 0007 South Africa; 3 Department of Statistics, University of Pretoria, Pretoria, South Africa

**Keywords:** Schizophrenia, Symptoms, Anxiety disorders, Classification, Diagnosis

## Abstract

**Background:**

Literature on anxiety in schizophrenia is confined to well-established diagnostic syndromes and the diagnostic category of unspecified anxiety disorder has not been quantitatively verified in this population. This study examined whether anxiety that is not differentiated into the well-established syndromes is empirically discernible from syndromal anxiety and no anxiety in acute-phase schizophrenia.

**Methods:**

After sampling 111 acute-phase schizophrenia patients, they were stratified into three groups: syndromal anxiety; undifferentiated anxiety; and without anxiety disorder. The groups were compared statistically in two data sets on measures for anxiety, psychotic severity, depressive features, akathisia and medication use.

**Results:**

On two measures of anxiety and for both data sets, the groups were significantly different without evidence of a confounding influence by akathisia, medication, or psychotic severity. The undifferentiated group was different from the syndromal group on the Staden Schizophrenia Anxiety Rating Scale (S-SARS) for both data sets (mean difference = 7.46, *p* < 0.001; mean difference = 7.69, *p* < 0.002) and on the Hamilton Anxiety Rating Scale for the one data set (mean difference = 14.68, *p* < 0.001) but not for the replicative data set (mean difference = 1.49, *p* = 0.494). The undifferentiated anxiety group was different from the no anxiety group for the respective data sets on both anxiety scales (S-SARS: mean difference = 8.67, *p* < 0.001; mean difference = 8.64, *p* < 0.001)(HAM-A: mean difference = 6.05, *p* < 0.001; mean difference = 8.67, *p* = 0.002). When depressive features had a confounding effect, it was small relative to the group differences.

**Conclusions:**

The results suggest some patients in acute-phase schizophrenia present with undifferentiated anxiety that is discernible from both syndromal anxiety and those without an anxiety disorder. This finding may serve as empirical grounds for clinicians to recognise undifferentiated anxiety in acute-phase schizophrenia, and for further research into the clinical importance of undifferentiated anxiety in this population.

## Background

Existing literature on anxiety in schizophrenia is confined to well-established diagnostic syndromes such as panic disorder, social anxiety disorder, specific phobias, obsessive–compulsive disorder, etc. [1–4]. It is not yet known whether anxiety that is not differentiated into these syndromes (that is, undifferentiated anxiety) is empirically discernible from syndromal anxiety and no anxiety in acute-phase schizophrenia. Undifferentiated anxiety is provided for in DSM-IV and DSM-5, respectively, as anxiety disorder not otherwise specified and unspecified anxiety disorder, which may be diagnosed concurrently with the diagnosis of schizophrenia [5, 6]. This nosological provision has not been quantitatively verified before in acute-phase schizophrenia.

Undifferentiated anxiety may be understood as a constellation of clinically significant anxiety features that do not meet diagnostic criteria for any of the typical anxiety syndromes. It may also be understood as an anxiety feature that is not specific to any one syndromal anxiety disorder. Palpitations, hypervigilance, restlessness, trembling, and feelings of tension are examples. In contrast, panic attacks, obsessions, compulsions, specific fears and excessive worries are more specific to the anxiety syndromes. One can thus distinguish between syndromal and undifferentiated anxiety, notwithstanding that syndromal and undifferentiated anxiety may co-occur. For example, it is conceivable that a patient may have clinical significant palpitations of anxiety, yet does not suffer from panic disorder, generalised anxiety disorder, or any of the other typical syndromes.

Prevalence rates of undifferentiated anxiety in schizophrenia are not known, but for syndromal anxiety the pooled prevalence rate of anxiety disorder was 38.3% in a meta-analysis of 52 studies, which was higher than the 28.8% reported for the general population [1, 7]. The pooled prevalence rates for the specific anxiety disorder were 12.1% for obsessive–compulsive disorder, 14.9% for social phobia, 10.9% for generalised anxiety disorder, 9.8% for panic disorder, and 12.4% for post-traumatic stress disorder. The prevalence rate of anxiety was similar in a recent study in which 45% of patients with schizophrenia had an anxiety disorder compared to 16% among controls [8].

Apart from schizophrenia, undifferentiated anxiety in the absence of syndromal anxiety was seemingly highly prevalent in the primary care setting. Seventy percent of anxiety disorder visits to primary care physicians were recorded as “anxiety state, unspecified” [9]. One may raise the question, however, whether undifferentiated anxiety is indeed so prevalent, or does this figure reflect a practice of these practitioners by which anxiety is simply not assessed and diagnosed in syndromal terms.

Undifferentiated anxiety in schizophrenia may compound the morbidity and mortality in schizophrenia as syndromal anxiety does. Syndromal anxiety has been found to impact negatively on quality of life [10, 11], functioning [11–14], overall psychopathology and the severity of comorbid medical conditions [15–18]. Increased rates of relapse, more frequent and longer duration of hospitalisations, poorer response to pharmacological treatments, substance abuse, negative attributional style, suicide and suicide attempts have been associated with anxiety in schizophrenia [19–25].

Anxiety may be more difficult to recognise during an acute phase of schizophrenia owing to the psychotic symptoms of this phase [26, 27]. Anxiety may further be clinically difficult to distinguish from the common extrapyramidal side effect of anti-psychotic medication, akathisia [3, 28]. Adding to this complexity, psychotic features and akathisia may exacerbate anxiety and vice versa [3, 26–28]. Similarly, comorbid depressive features correlate with anxiety both in the general [29, 30] and schizophrenia populations [31–34].

Considering these complexities in acute-phase schizophrenia [18, 34], research should account for the role of psychotic symptoms, akathisia and depressive features in verifying whether undifferentiated anxiety is empirically discernible from syndromal anxiety and no anxiety. Verifying undifferentiated anxiety in this population would warrant further research into its prevalence, contribution to morbidity, aetiology and treatment.

Given that anxiety may be more difficult to recognise in the presence of acute psychotic symptoms, this study aimed to account for this by examining particularly during an acute phase of schizophrenia whether undifferentiated anxiety presented symptomatologically distinct from syndromal anxiety and the absence of an anxiety disorder. Embedded in this aim, was to account for the potential role of psychotic severity, akathisia, medication and depressive features.

## Methods

### Study design

The study was designed to compare three groups in the acute phase of schizophrenia: (a) The undifferentiated anxiety group comprised patients who did not meet DSM-IV diagnostic criteria for syndromal anxiety, yet experienced undifferentiated anxiety. None of the DSM-IV diagnoses in the category of anxiety disorders applied to them except for anxiety disorder not otherwise specified; (b) The syndromal anxiety group comprised patients who all met DSM-IV diagnostic criteria for a syndromal anxiety disorder irrespective of having undifferentiated anxiety features concurrently. Any of the DSM-IV diagnoses in the category of anxiety disorders applied to them, except for anxiety disorder not otherwise specified; and (c) the no anxiety group comprising patients who did not meet DSM-IV diagnostic criteria for an anxiety disorder, nor for anxiety disorder not otherwise specified.

The three groups were stratified for diagnosis after convenience sampling. This means that analytical stratification was used and not stratification sampling, which reduced the risk for biased sampling and aided representation of patients with acute-phase schizophrenia, without anxiety features impacting on the sampling.

The stratification of the groups was done by two means: either directly through the Structured Clinical Assessment for DSM-IV Axis I Disorders (SCID) [35, 36], or indirectly through statistical modelling based on the SCID for participants who did not have the SCID performed as their data originated from an earlier stage of the research project. The former participants contributed accordingly to the SCID data set, whereas the latter participants contributed to a replicative data set. Comparisons among the groups had first been performed using the SCID data set, and then replicated for a second data set. The stratification of the replicative data set was done by extracting a discriminant analytic model of variables from the SCID data set that best predicted the three diagnostic groups in this data set. The model so derived from the SCID data set was then applied to the replicative data set for stratification into the three groups.

### Participants

Inclusion criteria were as follows: patients had to be between the ages of 18 and 65 years; they had to meet the diagnostic criteria of schizophrenia in an acute phase as defined by DSM-IV [5]; the acute-phase threshold had to be met by scoring more than 60 on the Structured Clinical Interview for the Positive and Negative Syndrome Scale (SCI-PANSS) [37]. In addition, they must have scored at least four (i.e., moderate) on any two of the SCI-PANSS items that constitute a psychotic item, namely those that measure hallucinatory behaviour, delusions, conceptual disorganisation, or suspiciousness; and the patients had to be able to give informed consent to participate in the study, affirming their informed consent by signing an informed consent document.

Patients were excluded from participation when they suffered from neurological conditions affecting the central nervous system; suffered from a known other medical condition that might influenced their mental state; suffered from alcohol dependence or dependence on another substance (excluding nicotine) according to DSM-IV criteria, or were likely to suffer from substance withdrawal symptoms; had taken a benzodiazepine less than 24 h prior to applying the measures used in this study; or had received zuclopenthixol acetate less than 72 h prior to applying the measures of this study. These exclusions averted potential confounding influences of these factors on anxiety.

All 111 participants were in-patients of Weskoppies Psychiatric Hospital in Pretoria, South Africa, who had been admitted to hospital for less than 10 days at the time of enrolment in the study. In the SCID data set on 60 patients, 22 were grouped in the undifferentiated anxiety, 20 in the syndromal anxiety and the remaining 18 in the no anxiety category. For replicative data set on 51 patients, the stratification model grouped 15 in the undifferentiated anxiety, 23 in the syndromal anxiety and 13 in the no anxiety category.

Written approval for conducting the research was granted by the Faculty of Health Sciences Research Ethics Committee of the University of Pretoria. Each participant gave informed consent to participate in the study, affirmed by signing an informed consent document. The study adhered to the 2013-version of the Declaration of Helsinki.

### Measures and variables

Two diagnostic instruments were used. The SCID was applied to stratify the patients into the three groups after sampling. The SCID and the Structured Clinical Interview for the Positive and Negative Syndrome Scale (SCI-PANSS) were used to ensure that patients were in the acute phase of schizophrenia with active psychotic symptoms [37]. The SCI-PANSS was also used as measure of the severity of the psychotic episode.

Anxiety was measured by two scales, for which measures of internal reliability in the acute-phase schizophrenia population are reported below. The Hamilton Anxiety Rating Scale (HAM-A) is one of the most established and widely used observer-rated scales for anxiety [38]. It comprises 14 items, each of which is rated from zero (i.e., none) to four (i.e., severe, grossly disabling). The items are mostly about bodily and behavioural manifestations of anxiety. The HAM-A is a general measure of anxiety that is not specific to any particular anxiety syndrome, or to schizophrenia. The Staden Schizophrenia Anxiety Rating Scale (S-SARS) is an observer-rated scale of anxiety specifically developed for use in schizophrenia patients [26, 27]. Each of ten items has six descriptive anchor points according to severity, scored from zero to five. The five items of the specific anxiety subscale assess persecutory and nihilistic anxiety, perceptual anxiety, anxiety attacks, situational anxiety and obsessive–compulsive anxiety. The general anxiety subscale comprises items on somatic anxiety, psychomotor and cognitive agitation, worry and fear, control-related anxiety and impairment from anxiety.

Depressive symptoms were measured by the Calgary Depression Scale for Schizophrenia (CDSS), a scale developed specifically for schizophrenia patients [39, 40]. Akathisia was measured by the Barnes Akathisia Scale (BAS) [41].

Other variables included age, gender, number of previous hospital admissions, and medication use in the categories of oral antipsychotics; depot antipsychotics during the preceding month; antidepressants; anticholinergic agents; benzodiazepines within the week of the interview (but not permissible within 24 h of applying the scales); mood stabilisers; and other medication.

### Statistical analyses

Comparisons had first been made across all three groups (undifferentiated anxiety, syndromal anxiety and no anxiety) with respect to the different scales. In the case of statistically significant results, subsequent pairwise comparisons of the undifferentiated anxiety group with the syndromal group and the no anxiety group were performed.

Comparisons across groups used the standard one-way analysis of variance (ANOVA) with bootstrapping, while independent samples *T*-tests, also employing bootstrapping, were performed post hoc with a Bonferroni adjustment for multiple testing to control for a Type I error.

Bootstrapping was used to resample randomly 1000 samples with replacement from a set of data points to estimate the precision of sample statistics. Bootstrapping was chosen owing to the sample sizes of both data sets, which were not large enough to warrant the assumption that a normal distribution would be appropriate for statistical tests based on the means. The *p*-values and confidence intervals obtained from a bootstrapped ANOVA is considered as “distribution-free” since they only rely on the data without making distributional assumptions.

If groups were statistically significantly different in relation to psychotic severity, depressive features, akathisia, or current medication, further analyses were conducted to examine whether these were confounders to the differences in anxiety found among the groups. To this end, analysis of co-variance (ANCOVA) with bootstrapping was performed across the three groups. The ANCOVA thus informed on the statistical significance of a difference in anxiety among the groups when accounting for the influence of covariates as well as on the statistical significance of the influence of a covariate. Moreover, it provided an estimated regression coefficient and its bias-corrected and accelerated (BCa) 95% confidence intervals that explain the extent of influence by a covariate in the group comparisons. For purposes of performing the ANCOVAs, the assumption of homogenous variances did not hold for the HAM-A analyses in the SCID data set, hence Brown–Forsythe and Welch corrections were made to validate the results [42, 43].

The probability of a Type I error—that is, to find a statistical difference when in fact there is no difference—was set at the conventional 5%. In addition to the bootstrapping utilised in performing ANOVA and ANCOVA to compensate for the sample sizes of the two data sets, statistical power calculations were performed to consider the chance of a Type II statistical error—that is, failing to reject the null hypothesis when it should have been rejected, or applied here, to have found no statistical difference when there was in fact a difference. Assuming an effect size (*f*) of 0.4 [44], and an *α* of 0.05, using the sample sizes of 60 and 51, in three groups with two degrees of freedom each, the power was calculated to be 0.78 for the SCID data set and 0.70 for the replicative data set. This means that the SCID and the replicative data sets were, respectively, subject to a 22% and a 30% chance of a Type II error.

In developing a model for the stratification of the replicative data set, the canonical discriminant analysis on SCID data set yielded two statistically significant factors (*p* < 0.001; *p* = 0.005), respectively, with eigenvalues of 3.7 and 0.56, and canonical correlation coefficients of 0.86 and 0.6, accounting for 86.8% and 13.2% of the variance. The model so derived correctly classified 88.3% of the patients into the three groups as classified by the SCID.

## Results

The age, gender, and number of previous admissions of the 111 participants in the two data sets are provided in Table 1. Table 2 presents the descriptive statistics (minimum, maximum, mean and standard deviation (SD)) for the scales as well as results on the Cronbach alpha (Cα), which is a measure of the internal consistency of items within each scale. The psychotic features were of a rather severe degree in both data sets as suggested by the SCI-PANSS mean scores of, respectively, 103.43 (SD = 13.87) and 97.61 (SD = 17.83), considering that a score of 60 is usually taken as the minimum threshold to constitute a psychotic episode. The internal reliability of the scales was good to excellent as suggested by the Cα values being close to and above 0.7.

**Table 1:** Demographic characteristics of participants

	SCID data set (*n* = 60)		Replicative data set (*n* = 51)	
Age	Minimum	19	Minimum	19
	Maximum	60	Maximum	64
	Mean	33.75	Mean	37.02
	SD	10.38	SD	13.29
Gender	Male	80%, *n* = 48	Male	76.5%, *n* = 39
	Female	20%, *n* = 12	Female	23.5%, *n* = 12
Previous admissions	Minimum	0	Minimum	1
	Maximum	14	Maximum	10
	SD	2.71	SD	2.55

**Table 2:** Descriptive statistics for the scales

Measures	SCID data set (*n* = 60)			Replicative data set (*n* = 51)		
	Mean	SD	Cα	Mean	SD	Cα
HAM-A	10.95	10.68	0.93	11.29	6.68	0.77
S-SARS						
Specific anxiety subscale	5.72	4.67	0.69	8.80	5.51	0.75
General anxiety subscale	8.87	5.54	0.85	10.08	5.12	0.79
Total score	14.58	9.62	0.88	18.88	10.13	0.88
CDSS	5.05	5.81	0.91	6.45	5.50	0.89
SCI-PANSS	103.43	13.87	0.75	97.61	17.83	0.86
Barnes Akathisia Scale	0.5	1.2	0.84	1.67	1.96	0.70

Table 3 reports the mean values and their 95% confidence intervals for scores obtained by the scales for the stratified groups. Statistically highly significant differences across all three groups were found on the HAM-A scale for both the SCID (*F* = 53.75, *df* = 2, *p* < 0.001) and replicative data sets (*F* = 53.75, *df* = 2, *p* < 0.001). On the S-SARS, the groups were also statistically significantly different for both data sets (SCID: *F* = 67.99, *df* = 2, *p* < 0.001); replicative: *F* = 63.01, *df* = 2, *p* < 0.001). As reported in Table 4, highly statically significant differences were found on the anxiety measures in all but one of the subsequent pairwise comparisons of the undifferentiated anxiety group with, respectively, the syndromal and the no anxiety groups.

**Table 3:** Means and 95% confidence intervals for the stratified groups

Measures	SCID data set (*n* = 60)			Replicative data set (*n* = 51)		
	Undifferentiated anxiety *n* = 22	Syndromal anxiety *n* = 20	No anxiety *n* = 18	Undifferentiated anxiety *n* = 15	Syndromal anxiety *n* = 23	No anxiety *n* = 13
HAM-A	7.7 (6.2–9.7)	22.5 (18.3–26.7)	1.7 (0.95–2.53)	12.9 (9.8–16.1)	13.9 (11.4–16.7)	4.7 (1.5–7.3)
S-SARS	14.3 (12.0–16.9)	24.3 (21.7–27.2)	4.2 (2.6–6.0)	17.5 (15.4–19.8)	25.8 (22.9–18.8)	8.2 (5.7–10.8)
CDSS	4.09 (2.1–6.8)	9.9 (7.3–12.6)	0.8 (0.3–1.5)	6.2 (3.8–8.6)	9.3 (7.1–11.5)	1.7 (0.5–3.2)
SCI-PANSS	106.4 (100.9–111.5)	104.6 (97.6–111.8)	98.6 (92.9–104.7)	96.1 (88.0–102.8)	107.5 (101.5–114.1)	81.9 (72.1–88.4)
BAS	0.4 (0.1–0.8)	0.9 (0.3–1.7)	0.2 (0–0.5)	1.3 (0.7–2.0)	2.3 (1.5–3.3)	0.9 (0.25–1.8)

**Table 4:** Pairwise comparisons of the undifferentiated anxiety with the syndromal and the no anxiety groups

Groups	SCID data set		Replicative data set	
	HAM-A	S-SARS	HAM-A	S-SARS
Syndromal anxiety vs. undifferentiated anxiety				
Mean difference	14.68	7.46	1.08	8.03
BCa 95% confidence intervals	10.34–9.21	3.73–10.98	− 2.94–4.93	4.52–12.08
Significance	*p* < 0.001**	*p* < 0.001**	*p* = 0.627	*p* < 0.001**
Undifferentiated anxiety vs. no anxiety				
Mean difference	6.05	8.67	8.22	9.31
BCa 95% confidence intervals	4.16–8.09	5.62–11.58	3.90–12.71	6.08–12.37
Significance	*p* < 0.001**	*p* < 0.001**	*p* = 0.002**	*p* < 0.001**

** Statistically highly significant

Akathisia was not statistically significantly different among the three groups in each of the data sets (SCID: *F* = 1.93, *df* = 2, *p* = 0.16; replicative: *F* = 2.52, *df* = 2, *p* = 0.091). Regarding prescribed medication, no statistically significant differences were found among the groups for both data sets.

There were no significant statistical differences in the severity of psychotic features among the three groups on the SCI-PANSS for the SCID data set (*F* = 1.72, *df* = 2, *p* = 0.189). For the replicative data set, a significant difference for the total SCI-PANSS scores among the three groups was found (*F* = 12.79, *df* = 2, *p* < 0.001), but on further analysis (i.e., ANCOVA), severity of psychotic features was not found to be a statistically significant covariate (regression coefficient = − 0.06 with 95% CI − 0.16 to 0.05; *p* = 0.241).

Depressive features were found to be significantly different among the three groups in both data sets (SCID: *F* = 19.58, *df* = 2, *p* < 0.001; replicative: *F* = 11.24, *df* = 2, *p* < 0.001). This prompted the examination of depressive features as a potential confounder by performing ANCOVAs with depressive features as the covariate. As reported in Table 5, no statistically significant confounding effect of depressive features was found in the comparison of the HAM-A scores among the groups in the replicative data set, but confounding effects of depressive features were found for the HAM-A in the SCID data and the S-SARS in both data sets. Although these were statistically significant, the confounding effects were relatively small as indicated by the regression coefficients. The regression coefficients that explain the effect of depressive features are all less than 0.5, which are thus markedly smaller than the regression coefficients that explain the group differences on the anxiety measures ranging from 4.44 to 17.61. Also the upper values of the BCa 95% confidence intervals for the regression coefficients that explain the effect of the depressive features are far from the lower values of the BCa 95% confidence intervals for the regression coefficients that explain the group differences on the anxiety measures.

**Table 5:** Relative effects of depressive features in the group comparisons

Dependent variable	Independent variable	Data set	Bootstrapped significance	Regression coefficient	BCa 95% confidence interval
HAM-A	Depressive features as covariate	SCID	*p* = 0.041*	0.49	0.02–0.91
		Replicative	*p* = 0.422	0.14	− 0.25 to 0.48
	Syndromal anxiety group	SCID	*p* = 0.001*	16.25	10.94–22.49
		Replicative	*p* = 0.002*	9.30	4.95–14.56
	Undifferentiated anxiety group	SCID	*p* = 0.002*	4.44	2.10–6.92
		Replicative	*p* = 0.002*	8.22	3.90–12.71
S-SARS	Depressive features as covariate	SCID	*p* = 0.01*	0.44	0.09–0.79
		Replicative	*p* = 0.008*	0.45	0.13–0.80
	Syndromal anxiety group	SCID	*p* = 0.001*	16.13	12.28–19.74
		Replicative	*p* = 0.001*	17.61	13.35–22.2
	Undifferentiated anxiety group	SCID	*p* = 0.001*	8.66	5.61–11.58
		Replicative	*p* = 0.001*	9.31	6.08–12.37

The no anxiety group is set to zero

* Statistically significant

## Discussion

This study found that some patients in acute-phase schizophrenia presented with undifferentiated anxiety that was discernible from both syndromal anxiety and the absence of anxiety. It is the first quantitative empirical verification of anxiety disorder not otherwise specified (DSM-IV) [5], called unspecified anxiety disorder in DSM-5 [6], in specifically acute-phase schizophrenia.

Empirical data showed that undifferentiated anxiety was discernible from no anxiety on two measures of anxiety. Undifferentiated anxiety was also discernible from syndromal anxiety on the S-SARS for both data sets and on the HAM-A for the SCID data set but not for the replicative data set. A reason for this inconsistency in the replicative data set may attributed to the HAM-A being dominated by undifferentiated anxiety rather than items specific to syndromes. A related reason may be that some patients with syndromal anxiety did not only have syndromal anxiety, but also undifferentiated features (as determined by the SCID). One may thus anticipate that the HAM-A, by addressing mostly undifferentiated anxiety, would not distinguish between these groups. It might have done, had the syndromal group been sub-stratified into those with and those without undifferentiated features. The difference of results between the data sets in this regard may relate to the fact that the stratification used for the replicative data set was based on the modelling of the SCID data. One may hypothesise that the modelled stratification used for the replicative data set had one of two effects: It uncovered the partial inability of the HAM-A to distinguish between the syndromal and the undifferentiated groups. Alternatively, the modelled stratification may have obscured the abilities of the HAM-A to distinguish between these groups. The latter hypothesis may be more persuasive considering that results from stratification using the SCID may trump results from stratification based on a statistical model. It may trump thus for two reasons: the empirical principle by which observed stratification is more persuasive than modelled stratification; and there were more participants in the SCID data set.

Undifferentiated anxiety was found discernible without evidence of a confounding influence by akathisia, the severity of psychotic symptoms, or medication. It was important to examine akathisia as potential confounder as it may be confused with anxiety and vice versa [3, 28, 45, 46]. Similarly, it was important to examine the potential influence of psychotic severity, owing to a previously reported relationship by which the severity of psychotic episode predicted more anxiety [26] and concerns about symptom differentiation that prompted a call to account for psychotic severity in anxiety studies in this population [27]. Our and other studies, furthermore, confirmed the presence of anxiety symptoms during the acute phase among some patients, toppling the outdated notion that anxiety symptoms in schizophrenia would be limited to the post-psychotic period [18, 31, 45].

Examining the influence of medication on the comparisons was important because it is well-established that medication may increase or decrease anxiety, even though the effects of anti-psychotic medication on anxiety have not been established conclusively as yet [47, 48]. The difference in anxiety among the three groups was not attributed to medication, but considering the relatively small sample size and the variety of medication, a Type II statistical error could not be excluded. Although not confounding the main finding of the study, medication might nonetheless have reduced the severity of anxiety across the groups, through direct physiological pathways and indirectly by the treatment of psychotic experiences that might in part have caused the anxiety [27, 49].

Depressive features had a small confounding effect in distinguishing undifferentiated anxiety from syndromal anxiety and the absence of anxiety, yet the statistical differences remained highly significant. The small confounding effect could be expected considering the well-known correlations between anxiety and depressive features generally [29, 33] and in schizophrenia [31–34].

The verification that undifferentiated anxiety was discernible from both syndromal anxiety and the absence of anxiety may serve as empirical foundation for clinicians to recognise undifferentiated anxiety in acute-phase schizophrenia. Patients’ undifferentiated anxiety may go unnoticed and may consequently be left untreated if clinicians are unaware that some schizophrenia patients present with discernible anxiety even though they do not suffer from any one of the typical anxiety syndromes. The clinical difficulties and complexities to distinguish anxiety features from schizophrenia symptoms have been noted before [1, 27, 34], but accounting for these practical difficulties and complexities begins with recognising them rather than conflating them as if all are the same. This may require clinical vigilance and deliberate diagnostic action, particularly because the psychotic symptoms in severe degrees of acute-phase schizophrenia (and the undoubted clinical challenges that these symptoms pose), may overshadow if not obscure the presence and importance of anxiety.

Clinical recognition of undifferentiated anxiety, as for syndromal anxiety, should be informed by appropriate screening and evidence-based integrated treatment approaches for anxiety symptoms beyond treating psychotic symptoms alone [3]. To this end, further research is required into the prevalence, causes, risk factors, epidemiology, course, outcomes, associated morbidity and efficacious treatments of undifferentiated anxiety.

The convenient sampling in our study does not allow for accurate prevalence figures, but pending research into this, our study suggests tentatively as follows: after sampling the one data set, as informed by the SCID, 36% of patients had undifferentiated anxiety in the absence of a typical anxiety syndrome, whereas about 33% patients had syndromal anxiety. This suggests that undifferentiated anxiety in acute-phase schizophrenia may be more common than any one of the typical anxiety syndromes, and may be about as prevalent as all the typical anxiety syndromes together.

The extent to which undifferentiated anxiety contributes to a poorer outcome and increased psychosocial impairment found for schizophrenia patients with anxiety disorders, needs to be researched, since there is growing evidence that comorbid anxiety has a negative impact on patients’ recovery and functioning [10–14, 50, 51]. Recognising and treating comorbid undifferentiated anxiety, as for syndromal anxiety [51–60], should achieve improved outcomes.

Undifferentiated anxiety may erode health-related quality of life as various forms of trauma individually and cumulatively do in schizophrenia [61]. Research into this will respond to the call that general psychopathology, and anxiety in particular, be researched in more detail to further clarify the factors involved in quality of life in schizophrenia [10–12]. Further research should also be undertaken in the hope of curbing preventable mortality in schizophrenia in as much as undifferentiated anxiety may contribute to suicide [23–25] and adverse cardiovascular effects of an increased heart rate and blood pressure when patients are acutely psychotic [62].

## Limitations

The results are confined to the acute phase of schizophrenia, averting the previously identified problems of heterogeneity of patient samples that included not only schizophrenia but also schizoaffective and psychotically depressed patients; the inclusion of patients at different stages of illness (for example, acutely psychotic, chronic outpatients, etc.); and inconsistent diagnostic assessments [63]. Participants in our study were severely ill in terms of their psychotic features, meaning that the results pertain unequivocally to the acute phase of schizophrenia, but whether our results pertain in the post-psychotic or inter-episodic phase of schizophrenia is yet to be established in a subsequent study.

The SCID for the DSM-IV was used and not its subsequent SCID-5-CV based on the DSM-5, which was published in 2016 after our study had already commenced [64]. This has no implication for the diagnosis of acute-phase schizophrenia, since changes relating to this disorder are rather editorial and not substantive. Nor are there implications for the change of “anxiety disorder not otherwise specified” in DSM-IV to “unspecified anxiety disorder” in DSM-5, which defined our study’s undifferentiated anxiety group. However, our study included obsessive–compulsive disorder and post-traumatic stress disorder among the syndromal anxiety disorders, whereas these two disorders were removed from the anxiety disorders category in DSM-5. Our inclusion of these two disorders among syndromal anxiety disorders remains justified inasmuch as dominant anxiety features are associated with these disorders.

It is a limitation that the SCID was not administered to participants of the replicative data set. Had this been done, a single set of analyses for participants of both data sets would have been possible. Instead, a statistically modelled stratification was followed by which the second data set could be made useful, strengthening the design beyond the one data set and providing for the advantage of performing two sets of analyses independently.

The sample size of 111 is a limitation. A small sample size is particularly relevant to a Type II statistical error—that is, to retain incorrectly a false null hypothesis. All the comparisons for which no statistically significant difference was found, were thus potentially subject to a Type II error. The finding of no statistical difference in the replicative data set on the HAM-A between undifferentiated and syndromal anxiety groups is an example. Restricted to these findings of no statistical difference, the chance of a Type II error was found to be 22% for the SCID data and 30% for the replicative data set based on post hoc power calculations and their embedded assumptions. This is acceptable considering that sample size calculations are typically performed using a power of 80%, i.e., a chance of a Type II error of 20%.

Being a statistically significant finding, a potential Type II error was not relevant to the main finding of the study by which some patients in acute-phase schizophrenia presented with undifferentiated anxiety that was discernible from both syndromal anxiety and the absence of anxiety. In contrast, the main finding was subject to a potential Type I error, which applies to the significant findings. The risk of a Type I error was mitigated by using two data sets as well as statistical tests that did not depend on distributional assumptions or a large sample size but compensated for the smaller than ideal sample size by employing the resampling technique of bootstrapping.

The study was by design limited to the empirical clustering of symptoms and signs. It did not extend to an investigation into causes, risk factors, epidemiology, course, morbidity, or outcomes. Studies on the causes of anxiety in schizophrenia, for example [65, 66], may be advanced by distinguishing between undifferentiated and the specific anxiety syndromes. Our study may be seen as a precursor to such investigations that may explain the patterns in which the anxiety symptoms and signs clustered in our study.

## Conclusions

This first quantitative verification of undifferentiated anxiety provides empirical substance to DSM-IV and DSM-V provisions for undifferentiated anxiety as anxiety disorder not otherwise specified and unspecified anxiety disorder, respectively, in the specific population of acute-phase schizophrenia. Undifferentiated anxiety was found to be discernible from syndromal anxiety without evidence of a confounding influence by the severity of acute-phase schizophrenia symptoms, akathisia, or medication. When depressive features had a confounding influence, the influence was relatively small. The main finding of this study may serve as empirical grounds for clinicians to recognise undifferentiated anxiety in acute-phase schizophrenia. It may also serve as a grounds for further research into the clinical importance of undifferentiated anxiety in this population.

## Data Availability

The datasets used and analysed during the current study are available from the corresponding author on reasonable request.

## Abbreviations

ANCOVA: Analysis of co-variance
ANOVA: Analysis of variance
BCa 95% CI: Bias-corrected and accelerated 95% confidence intervals
Cα: Cronbach alpha coefficient
CDSS: Calgary Depression Scale for Schizophrenia
DSM-IV: Diagnostic and statistical manual of mental disorders—fourth edition
DSM-5: Diagnostic and statistical manual of mental disorders—fifth edition
HAM-A: Hamilton Anxiety Scale
SCID: Structured Clinical Assessment for DSM-IV Axis I Disorders
SCI-PANSS: Structured Clinical Interview for the Positive and Negative Syndrome Scale
SD: Standard deviation
S-SARS: Staden Schizophrenia Anxiety Rating Scale
